# Molecular characterization of Peste des Petits ruminants virus isolated from four outbreaks occurred in southern Iran

**DOI:** 10.1186/s12917-019-1920-y

**Published:** 2019-05-28

**Authors:** Reza Shahriari, Azizollah Khodakaram-Tafti, Ali Mohammadi

**Affiliations:** Department of Pathobiology, School of Veterinary Medicine, Shiraz University, Shiraz, Iran

**Keywords:** Peste des Petits ruminants virus, N gene, Phylogenetic tree

## Abstract

**Background:**

Peste des petits ruminants (PPR) is a severe infectious disease in both domestic and wild small ruminants. Due to its heavy economic burden and hence social and health impacts on human populations, Food and Agriculture Organization of the United Nations (FAO) and The World Organization for Animal Health (OIE) have targeted PPR for eradication by 2030. In order to plan and implement a successful eradication program, factual status assessments prior to devising disease control strategies is a vital criterion. The aim of this study was to isolate and characterize PPR virus from a rising wave of outbreaks in southern Iran.

**Results:**

Twenty-one clinical samples, including blood as well as oral, nasal and ocular swabs were collected from ten sick animals in 4 various herds and were examined with ELISA and RT-PCR for the presence of PPR virus antigen and genome, respectively. The virus was successfully isolated in primary lamb kidney cell culture and identified by RT-PCR. Phylogenetic analysis of the sequenced N genes revealed that, while the earliest reports of Iran’s outbreaks were grouped into clusters with Saudi Arabia, Turkey and Africa, in this study reported sequences were grouped with samples from Pakistan, Tajikistan and China in particular. This observation suggests a shift in PPRV flow from the western borders of the country to the eastern neighboring countries.

**Conclusions:**

Lineage IV of PPR virus is presently circulating in Iran, with certain levels of genetic diversity. Present study along with previous reports demonstrates the dispersal patterns and movements of PPR virus, which highlights the reversal pattern of virus flow in recent years. Such information is necessary to understand PPRV molecular epidemiology and to develop more proper control strategies to eradicate the disease in the planned time.

**Electronic supplementary material:**

The online version of this article (10.1186/s12917-019-1920-y) contains supplementary material, which is available to authorized users.

## Background

Peste des petits ruminants, also known as goat plague, is an acute highly contagious viral disease that causes serious economic losses due to high morbidity and mortality rates [1–3]. This disease affects mainly sheep and goats, sometimes wild small ruminants and camel [4, 5]. The causative agent, PPR virus, enveloped virus has a capsid and a single-stranded RNA genome that has been shown to be the largest member of the *Morbillivirus* genus [6]. The genome of PPR virus encodes 6 structural proteins, including a nucleoprotein (N), a viral RNA-dependent polymerase (L), an RNA-polymerase phosphoprotein co-factor (P), a matrix protein (M), a fusion protein (F) and a hemagglutinin protein (H). According to the sequence analysis of N, F or H genes of PPR virus strains, N gene has been proposed as the most appropriate gene for molecular characterization of closely related isolates. [7]. Based on molecular genotyping PPRV has been classified into four lineages [8], each with a specific geographical distribution pattern that has been changed in recent years. Lineage I were commonly found in West Africa and have recently been reported in central Africa, lineage II isolates are found in western Africa, lineage III in eastern Africa, and some parts of the Middle East and the lineage IV in the Arabian Peninsula, the Middle East and South Asia [8, 9]. Recent reports showed the emergence of PPR virus lineage IV in the northeast and North Africa [10]. While distinct virus geographical dispersal based on genotyping suggests its pre-existence long before identification in mentioned regions, recent reports of unexpected virus lineage introduction in regions previously associated with other lineages implicates the virus flow. Furthermore, recent occurrences of PPRV in disease-free areas demonstrates PPRV movements and its rapidly evolving epidemiological situation [11].

Located in Eurasia and western Asia, Iran is considered a crossroads of three continents, Asia, Europe, and Africa. According to the latest report of OIE Iran has 10 and 5% of the Middle East sheep and goats herd populations, respectively. Despite the use of the live attenuated vaccine, it is not unexpected that 32% of the reported outbreaks of PPRV over the last 16 years were from Iran with over 5000 outbreaks being recorded between 2005 and 2011. Since the first PPRV outbreak report in 1995 [12], till now most reports from Iran solely relied on either clinical outcomes or serology tests except one report with molecular characterization and phylogenetic analysis based on F gene [13].

Determining the nature of circulating strains of PPR virus has a crucial role in devising more appropriate diagnostics and control strategies. Therefore, the aim of the present study was to isolate, characterize and phylogenetically analyze the circulating PPR virus strains in small ruminants’ outbreaks in Fars province, Iran.

## Results

### Field observations

**Fig. 1:**
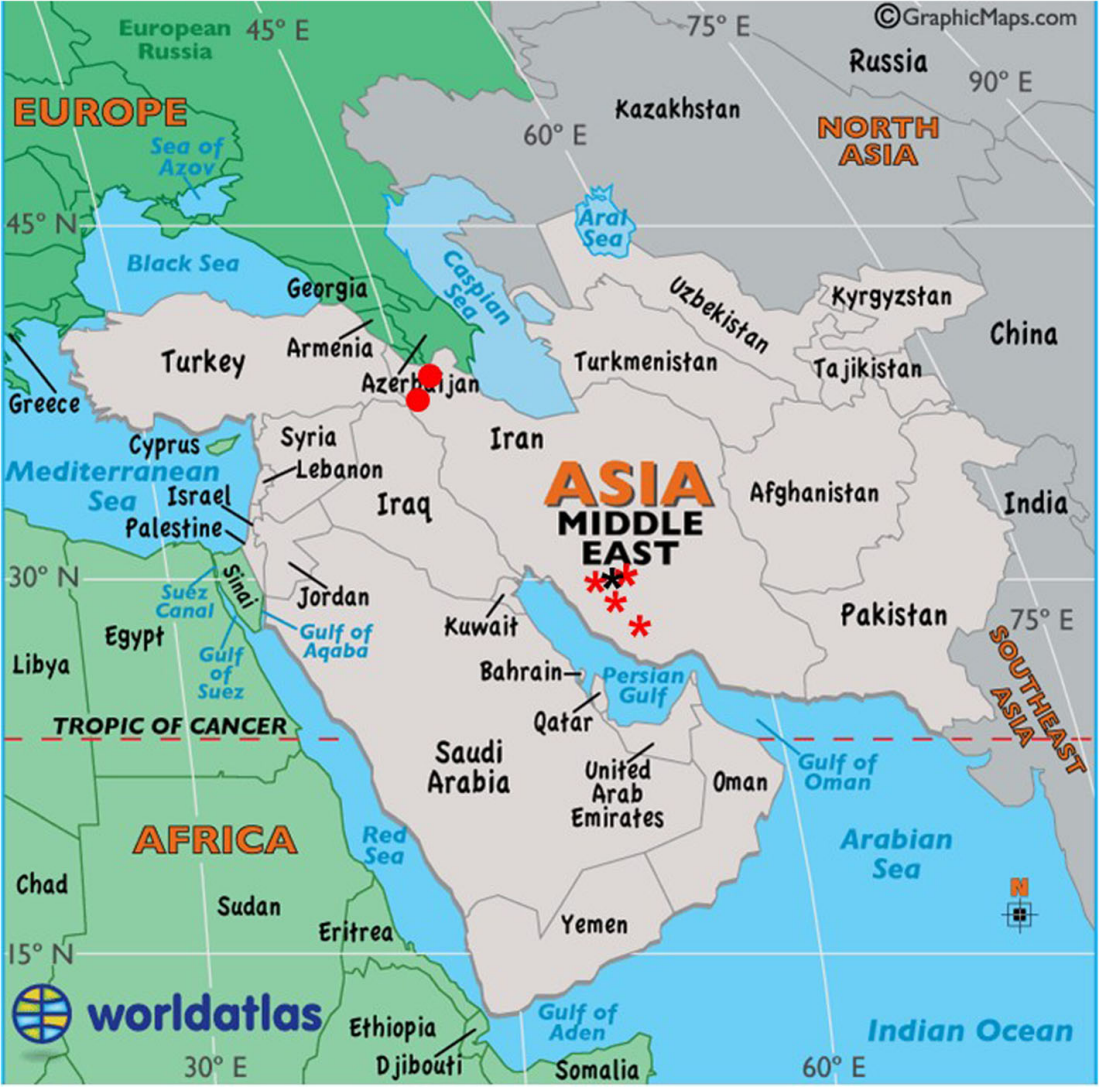
Geographical location of identified outbreaks. Four distinct outbreaks in close proximity in Fars province of Iran

The characteristic clinical signs of PPR were observed (Fig. 1) in affected animals including high fever (more than 40 °C), catarrhal nasal discharge, crusts around the nostrils, lacrimal discharge, erosive-ulcerative oral lesions covered with gray to yellow pseudomembrane on the gums, lips, tongue and palates (Additional file 1: Figure S1) and also diarrhea.

### Diagnostic assays results

All nasal and oral swabs submitted to the local veterinary service were reported positive for PPR virus antigen using ELISA. These results demonstrate appropriate clinical criteria were applied for the sample collection procedure. RT-PCR of samples also confirmed that all samples were positive for PPR. Further, for molecular detection, RT-PCR was conducted to amplify parts of N gene and to confirm this amplification, electrophoresis of RT-PCR products was performed and the expected band was observed at 352 bp.

### Virus isolation in primary fetal lamb kidney (PFLK) cell culture

**Fig. 2:**
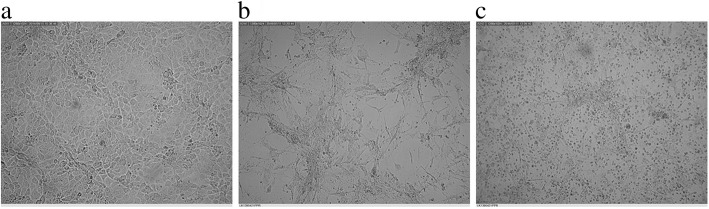
Virus isolation in primary cell culture. **a** Primary culture of lamb kidney cells. **b** Cells treated with PPR virus demonstrate syncytia formation. **c** Cell destruction twelve days post inoculation (magnification 100X)

Since virus isolation in cell culture is considered as the golden standard of virus diagnosis, virus isolation was carried out in primary lamb kidney culture. PPR viruses infected PFLK cell culture monolayers showed cell rounding 16 days post-inoculation (PI). The cytopathic effect (CPE) similar to a *Morbillivirus* infection was observed as aggregates of rounding cells and syncytia formation after nine days of PI, further, cells destruction occ twelve days of PI (Fig. 2).

#### Phylogenetic tree

**Fig. 3:**
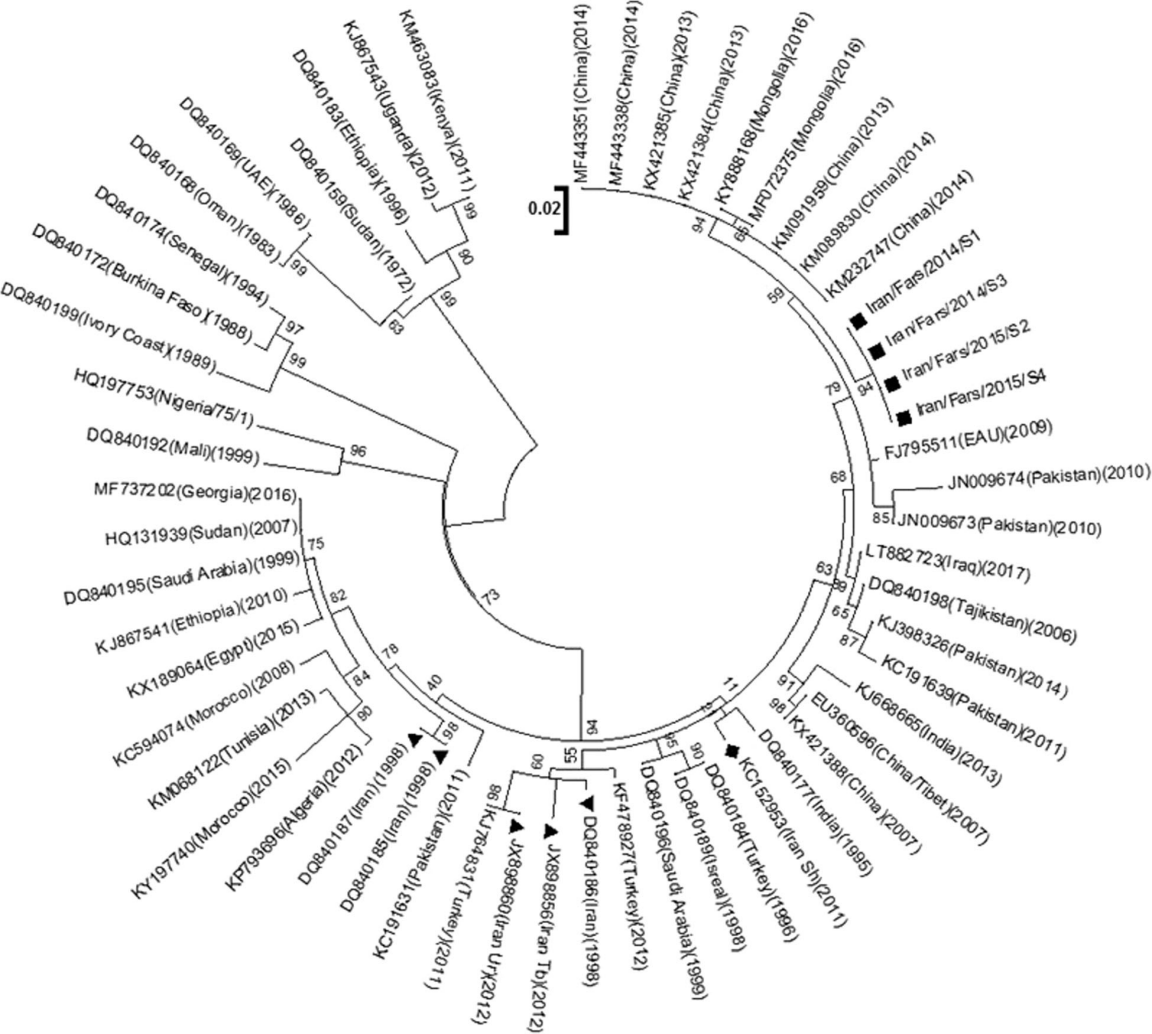
Phylogenic analysis of the PPRV. Maximum Likelihood consensus tree with Kimura two-parameter model in Mega7 version 5 at bootstrap value 1000 replicates based on PPRV partial N gene sequence (352 nt) isolated from four Iranian outbreaks (black square) (MH824412, MH824413, MH824414, MH824415) and selected sequences from GenBank (NCBI). In the figure, bootstrap values of only above 50% are shown. The horizontal lines were proportional to the distance among sequences

In order to represent a comprehensive picture of PPR epidemiology and its circulating strains in the region, multiple alignments and phylogenetic tree construction were performed using the obtained sequences in this study and a collected list of various sequences reported previously worldwide (Fig. 3). These analyses were based on N gene which has been previously identified as the best candidate for PPRV phylogenetic studies [7]. Besides, the attained 351 bp sequence of N genes in this study were submitted to GenBank with accession numbers of (MH824412, MH824413, MH824414 and MH824415). Maximum likelihood approach with the Kimura two-parameter model was applied to compare all mentioned sequences. As expected all samples of this study clustered together, which suggests a common strain accounting for all 4 outbreaks under survey. Surprisingly, these sequences were in close proximity to several reported sequences from China and Mongolia from 2013 to 2016 [14–16]. Previous sample of Fars province in 2011, Iran Sh (KC152953), clustered with India far from the present study sequences. It should be noted that two other reports of Iran in 2012, Iran Tb (JX898856) and Iran Ur (JX898860), are reports from the northwest of Iran which shares a common border with Turkey. Overall, all samples reported from Iran clustered into lineage IV which used to be exclusively prevalent in Asia but recently has expanded its distribution through other continents like Africa. The resulted tree also demonstrates that while initial reports of Iran were mostly clustered with sequences from Saudi Arabia and Africa, later reports were clustered with reports of Turkey, Tajikistan, Pakistan and recently with China.

## Discussion

Having 50% of the sheep herd population in the Middle East, Iran is accounted to be the 4th country in sheep herd population worldwide. Most of these small ruminants are either kept by nomad populations or rural villagers with constant trading in between, mostly at traditional and religious events. This situation makes a region vulnerable to recurrent outbreaks of contagious disease as is observed in the case of PPRV epidemics in Iran.

Since the first report of PRRV in Iran in 1995 [12] numerical outbreaks have occurred in this region so that over the recent 16 years, one-third of all PPR outbreaks’ reports to OIE were from Iran. Nevertheless, few surveys have been conducted to decipher the genetic nature and the molecular genotyping of circulating strains. In this study, we represent PPR virus isolation and characterization in four distinct outbreaks in Fars province of Iran.

In previous reports [17] sandwich-ELISA was reported to be positive for 71.9% of PPR suspected samples. In our study, 100% of suspected samples tested with sandwich-ELISA, were confirmed as positive. This high level of accuracy can be attributed to the strict clinical signs criteria adopted and demonstrates accurate field observation. These results also highlight the prevalence of PPR circulating virus in the region. Further, RT-PCR of collected samples also confirmed the ELISA results.

As a proper amount of virus genetic material was needed for further molecular characterization and sequencing, we conducted virus isolation on primary lamb kidney cell culture. Inoculated nasal and oral swabs taken from live affected animals were used for this purpose. So far, the typified phenotype of CPE of *Morbillivirus* infection in addition to subsequent syncytia formation and cell destruction has exhibited the successful isolation procedure.

In order to gain insight into the genetics nature of circulating virus in the region, we depicted a phylogenetic tree based on the N gene partial sequence. Recent studies have proved that N gene sequence can more accurately discriminate different strains in comparison to F and H genes. [7]

The phylogenic tree shows that the present analyzed PPR viruses are more closely related to reported strains from China, Tajikistan, and Pakistan. Furthermore, this analysis reveals distant clustering of reported sequences in this study with previous reports of this region The possible relationship of Iranian strains resemblance to that of Pakistani strains of PPR virus could be due to the fact that Iran has common borders with Pakistan and Afghanistan where uncontrolled animal trade across borders, along with trans boundary movements encompassing Pakistan, Afghanistan and Tajikistan is quite common. Yet close proximity of reported sequences in this study with recent reports of China, within a short period, suggests virus introduction through importing small ruminants from this country. The grouping of these strains of PPR virus at two different places of the phylogenic tree over time shows that PPR virus isolates may have undergone exceptional evolutionary pathway or this might be as a result of virus introduction from a variety of sources. Considering the latter possibility, this observation suggests a reversal pattern of virus flow in recent years. However, more samples collected from a wider range of outbreaks are needed to evaluate such commentary.

## Conclusion

In spite of being endemic in Iran, outbreaks of PPRV are regularly occurring, and limited information is available on the genetic nature of PPRV. The sequences described in this study increase the available information on the circulating strains of PPRV in Iran. With these bounded data, we still endeavor to demonstrate the presence of a new population of PPRV, which necessitates future such studies to be performed at the national level to determine the complete picture of circulating viruses in the country. According to the findings of this study, the circulation of the virus from the west to the east of Iran is now reversed, from the east to the west. Therefore, proper understanding of this virus circulation will help PPRV endemic countries such as Iran develop effective control strategies**.**

## Methods

### Sample collection

During a period of 14 months (June 2014 to August 2015), samples from twenty-one domesticated small ruminants (5 sheep and 16 goats) with clinical outcomes consistent with PPRV were collected in Fars province of Iran (Fig. 1). These samples were from four diverse waves of disease susceptible to PPR. All sampled animals were non-vaccinated. The clinical signs of the animals were recorded. A brief history of collected samples and clinical presentations in the suspected animal is provided in. Table 1. The total of samples including nasal and oral swabs, necrotic debris and uncoagulated blood in EDTA were collected from the affected animals. Each sample was labeled with a unique identifier as a specific number, date, and species. These samples were then stored in ice pack shipper and immediately transferred to the laboratory for further processing.

**Table 1:** Descriptive sample data collected for this study

Date	Location	Clinical sign	Place	Sample	Animals sampled/total animal in the herd
2014/6/25	Sarvestan	Fever(41.2 °C), serous oculonasal discharge, erosive stomatitis, coughing	Multi-farm complex	Nasal swab A swab of necrotic debris from buccal mucosa, blood in EDTA	6/320
2014/8/4	Zarin Dasht	Fever(40.7 °C), mucopurulent oculonasal discharge, necrotic stomatitis, coughing,	Nomad livestock	A nasal swab of necrotic debris from buccal mucosa, blood in EDTA	5/180
2015/7/7	Shiraz	Fever (41.4 °C), serous oculonasal discharge, erosive stomatitis, coughing,	Live animal market	A nasal swab of necrotic debris from buccal mucosa, blood in EDTA	5/65
2015/8/8	Zarghan	Fever (40.9 °C), mucopurulent oculonasal discharge, necrotic stomatitis, coughing	Fattening farm	A nasal swab of necrotic debris from buccal mucosa, blood in EDTA	5/200

Nasal and oral swabs, necrotic debris and uncoagulated blood in EDTA were collected from goats and sheep with PPRV consistent clinical signs

### Laboratory investigations

Sandwich-ELISA method was used for initial confirmation of PPRV antigen in the specimens in the local veterinary service lab. All samples were tested individually in duplicate according to the manufacturer’s instructions. The ELISA kit was purchased from ID vet (France) and detected N antigen, as described [18].

### Cell culture and virus isolation

PFLK was prepared in the Department of Virology at Shiraz University for the virus isolation. The cells were incubated at 37 °C with 5% CO2 and monitored daily for CPE caused by the virus. After observation of CPE similar to a Morbillivirus, cell rounding, and syncytia formation, the cells were frozen and thawed 3 times and centrifuged. The supernatants were used for RNA extraction.

### RNA extraction and RT-PCR and sequencing

All samples, as well as supernatant of destroyed cultured cells and PPRV vaccine (as the positive control), were subjected to RNA extraction using RNA-Plus Solution (CinnaGen®, Iran). RNA extracts were stored at − 70 °C until use. The swab obtained from the animal without any clinical sign and negative for Ic-ELISA was used as negative control.

The synthesis of cDNA of PPRV was performed according to the manufacturer’s procedures of AccuPower® Cycle Script RT PreMix (dN6) from all samples (Bioneer Corporation, Korea). RT products were cooled on ice and stored at − 20 °C until used.

For RT-PCR, oligonucleotide primers corresponding to N gene sequence were used. These primers were adopted from O.I.E. proposed primers with some modification. Upstream primer sequence: (NP4–19) 5’CCTCCTCCTGGTCCTCCAG3´. Downstream primer sequence: (NP3–17) 5’GTCTCGGAAATCGCCTC3´.This primer pair amplifies 352 bp region of N gene. The PCR was performed by AMPLICON master mix (Denmark) according to the manufacturer recommendation.

### Phylogenetic tree

Four samples of RT-PCR products representing N genes of isolated positive samples in cell culture were submitted to Macrogen Corporation for sequencing.

The electropherogram files of resulting sequences were examined and edited using Chromas. Partial N gene sequences generated in other reports were obtained from GenBank, Fig. 3. The nucleotide sequence alignments were carried out by Mega7. The phylogenetic tree was constructed by the maximum likelihood method with the Kimura two-parameter model at the bootstrap value of 1000 replicates.

## Additional file

Additional file 1: **Figure S1.** Ulcerative stomatitis in a PPRV affected goat. (JPG 58 kb)

## Data Availability

All authors are responsible to share the supporting data and materials upon reasonable requests. The details of the sequences were uploaded in GenBank and the accession numbers are MH824412, MH824413, MH824414 and MH824415.

## Abbreviations

CPE: Cytopathic effect
EDTA: *Ethylenediaminetetraacetic acid*
F: Fusion protein
FAO: Food and agriculture organization of the United Nations
H: Hemagglutinin
Ic-ELISA: Immunocapture enzyme-linked immunosorbent assay
INSF: Iranian National science foundation
L: Viral RNA-dependent polymerase
M: Matrix protein
N: Nucleoprotein
OIE: Office International des Epizooties (The World Organization for Animal Health)
P: RNA-polymerase phosphoprotein co-factor
PFLK: Primary fetal lamb kidney
PI: Post inoculation
PPRV: Peste des petits ruminants virus
RT-PCR: Reverse transcription-polymerase chain reaction
