# Supplementary figures and images for: Molecular characterization of Peste des Petits ruminants virus isolated from four outbreaks occurred in southern Iran
Source: BMC Vet Res. 2019 May 28;15:177. doi: 10.1186/s12917-019-1920-y (PMC6540375; doi:10.1186/s12917-019-1920-y)

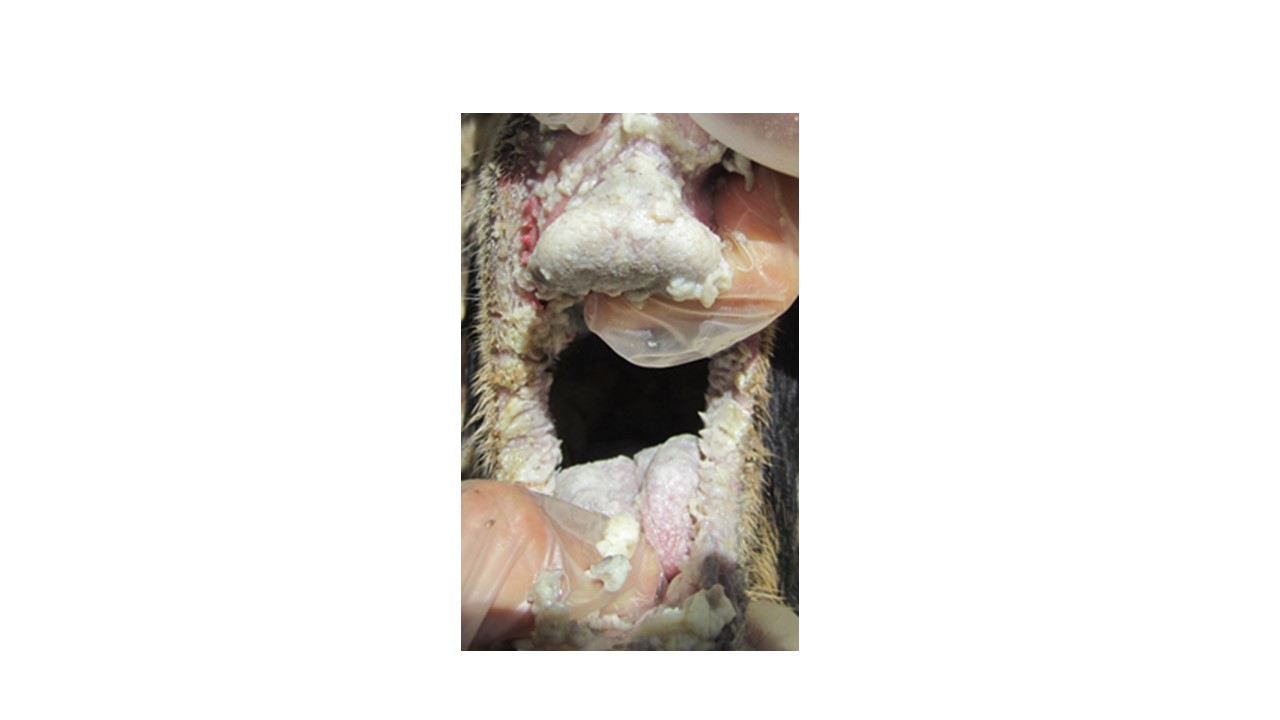

Supplement: Supplementary file 1 — Figure S1. Ulcerative stomatitis in a PPRV affected goat. (JPG 58 kb) [file 12917_2019_1920_MOESM1_ESM.jpg]
